# The Prevention of Venereal Disease

**Published:** 1917-06-09

**Authors:** 


### The Hospital

The Workers' Newspaper of Administrative Medicine and Institutional Life, Administration, National Insurance and Health.

No. 1618, Vol. LXII. SATURDAY, JUNE 9, 1917. PRICE ONE PENNY

### THE PREVENTION OF VENEREAL DISEASE.

The fate which often attends the reports of Royal Commissions has not awaited the Report of the Royal Commission on Venereal Diseases. So far from shelving the subject the Report has pushed it into the forefront and, aided by circumstances, it has succeeded not only in enlightening public opinion but in arousing a demand for action. What was formerly regarded as a forbidden topic is now recognised as suitable for public debate and dramatic representation. The facts are faced, and it is agreed that they be set forth in such a fashion that even he who runs may read. All this is to the good, as are also the practical measures which have come out of it. Among these, quite properly, emphasis has been laid on the provision of facilities for early and expert treatment, and it is beyond challenge that from the arrangements already made many individual sufferers will gain substantial benefit. Further, in so far as such measures tend to lessen the spread of the disease, they have a degree of preventive value, and public money spent on their establishment may thus be justified on the broadest possible grounds. Yet, important as is efficient treatment of the victims of these diseases it is a relatively small issue compared to the question of prevention. And on the question of prevention there is yet much education to be accomplished.

In certain quarters a good deal of stress is placed on the value of giving to the public a picture of the possible consequences of syphilitic or other venereal infection. Pamphlets and leaflets are put into circulation, and the stage takes up the gruesome tale. Society is thrilled, and much is hoped from these warning voices. It would not be safe to say that such methods have no efficacy, as it is at least highly probable that some persons will avoid risk from dread of the possible consequences. But though perhaps new in form these methods are not new in substance. In a less definite but still in a very real fashion there has been widely spread in the community a knowledge of the dangers which attend illicit sexual intercourse, and it may fairly be questioned whether the new emphasis will add much efficacy to the argument. There is, further, the chance of mischief which comes from misstatement, and of presenting as frequent and usual what is really only occasional and exceptional. Many good causes have been injured by the exaggerations of their advocates, and when wise counsel relies on such exaggerations it is apt to suffer complete wreck as the fallacious statements reveal their true character in the school of practical experience. It is not easy, we freely admit, to present the sober truth of the situation, and no medical practitioner would desire to minimise in any degree the risks and mischiefs of these diseases. But there is danger in the other direction and it is not being fully avoided. Reaction and incredulity may lead to a fuller licence, just as, we are told, the reign of the Puritans was followed by the reign of the harlots. We stand fully for education and knowledge, but we see the risk of exaggeration and we are satisfied that no new educational campaign can have such superior virtues to the older methods as to justify the belief that lectures and leaflets and sermons and stage plays will preach venereal diseases out of existence. The record of history is opposed to such an expectation.

While educational enlightenment and moral suasion form one line of policy there is another scheme which relies frankly on material methods. Teach and preach as much as you like, say its advocates, but in the end the question is one of preventive medicine and it must be dealt with from this point of view. Persons who expose themselves to risks of infection ought to be provided with the information and methods by means of which these risks may be neutralised or at least may be seriously lessened. In this way the number of persons infected will be greatly reduced and the opportunities for extension of disease in the community will be much curtailed. There is no doubt that such a claim may be supported by a not inconsiderable body of experience, for the issue has been put to the test on a fairly extensive scale. Certainly the proposal raises a very delicate ethical question for members of the medical profession, and though some general discussion on it has taken place, it has not been brought to an issue by the corporate bodies which in greater or less measure help to form professional opinion. The discussion is apt to pass from medicine to morals and to excite a good deal of feeling. But sooner or later the profession will have to make up its mind on the question, and any day commercial enterprise may take it in hand.

A more pleasing preventive measure is presented by the advocates of early marriages, who would fain re-establish the family ideals of an earlier day. And when the nation is losing so many of its youth on the battle-field the proposal has a specially attractive quality. It is quite possible that public opinion may do something in this direction and may restore Mr. Quiverful to his position as a model patriot. Were it put into operation, there can be no doubt that the practice would be a powerful factor in limiting the frequency of venereal diseases. But the method is not quite so simple as it sounds. A wide acceptance of it would mean much individual and social readjustment, and this both in opinion and in practice. This consideration, however, ought not to deter the advocates of the policy, and for our own part we feel for them a large measure of good-will.

The point which we wish to make in this brief review is that the true end of all publicity on this question is prevention, and it is to this end that public opinion ought to be directed. The circumstances of the moment would seem to justify a more active police crusade against the professional distributors of contagious diseases, but to think that here is the solution of the whole question is, we are convinced, a mere delusion. Something larger and more radical is needed if permanent and widespread effects are to follow.

